# UmetaFlow: an untargeted metabolomics workflow for high-throughput data processing and analysis

**DOI:** 10.1186/s13321-023-00724-w

**Published:** 2023-05-12

**Authors:** Eftychia E. Kontou, Axel Walter, Oliver Alka, Julianus Pfeuffer, Timo Sachsenberg, Omkar S. Mohite, Matin Nuhamunada, Oliver Kohlbacher, Tilmann Weber

**Affiliations:** 1 The Novo Nordisk Foundation Center for Biosustainability, Technical University of Denmark, Kemitorvet Building 220, 2800 Kgs. Lyngby, Denmark; 2 Applied Bioinformatics, Department of Computer Science, Eberhard Karls University Tübingen, Sand 14, 72076 Tübingen, Germany; 3 Institute for Bioinformatics and Medical Informatics, University of Tübingen, Sand 14, 72076 Tübingen, Germany; 4 Translational Bioinformatics, University Hospital Tübingen, Schaffhausenstr. 77, 72072 Tübingen, Germany; 5 Visual and Data-Centric Computing, Zuse Institute Berlin, Takustr. 7, 14195 Berlin, Germany; 6 Algorithmic Bioinformatics, Freie Universität Berlin, Takustr. 9, 14195 Berlin, Germany

**Keywords:** Untargeted metabolomics, Processing, Analysis, High-throughput workflow, Software

## Abstract

Metabolomics experiments generate highly complex datasets, which are time and work-intensive, sometimes even error-prone if inspected manually. Therefore, new methods for automated, fast, reproducible, and accurate data processing and dereplication are required. Here, we present UmetaFlow, a computational workflow for untargeted metabolomics that combines algorithms for data pre-processing, spectral matching, molecular formula and structural predictions, and an integration to the GNPS workflows Feature-Based Molecular Networking and Ion Identity Molecular Networking for downstream analysis. UmetaFlow is implemented as a Snakemake workflow, making it easy to use, scalable, and reproducible. For more interactive computing, visualization, as well as development, the workflow is also implemented in Jupyter notebooks using the Python programming language and a set of Python bindings to the OpenMS algorithms (pyOpenMS). Finally, UmetaFlow is also offered as a web-based Graphical User Interface for parameter optimization and processing of smaller-sized datasets. UmetaFlow was validated with in-house LC–MS/MS datasets of actinomycetes producing known secondary metabolites, as well as commercial standards, and it detected all expected features and accurately annotated 76% of the molecular formulas and 65% of the structures. As a more generic validation, the publicly available MTBLS733 and MTBLS736 datasets were used for benchmarking, and UmetaFlow detected more than 90% of all ground truth features and performed exceptionally well in quantification and discriminating marker selection. We anticipate that UmetaFlow will provide a useful platform for the interpretation of large metabolomics datasets.

**Graphical Abstract:**

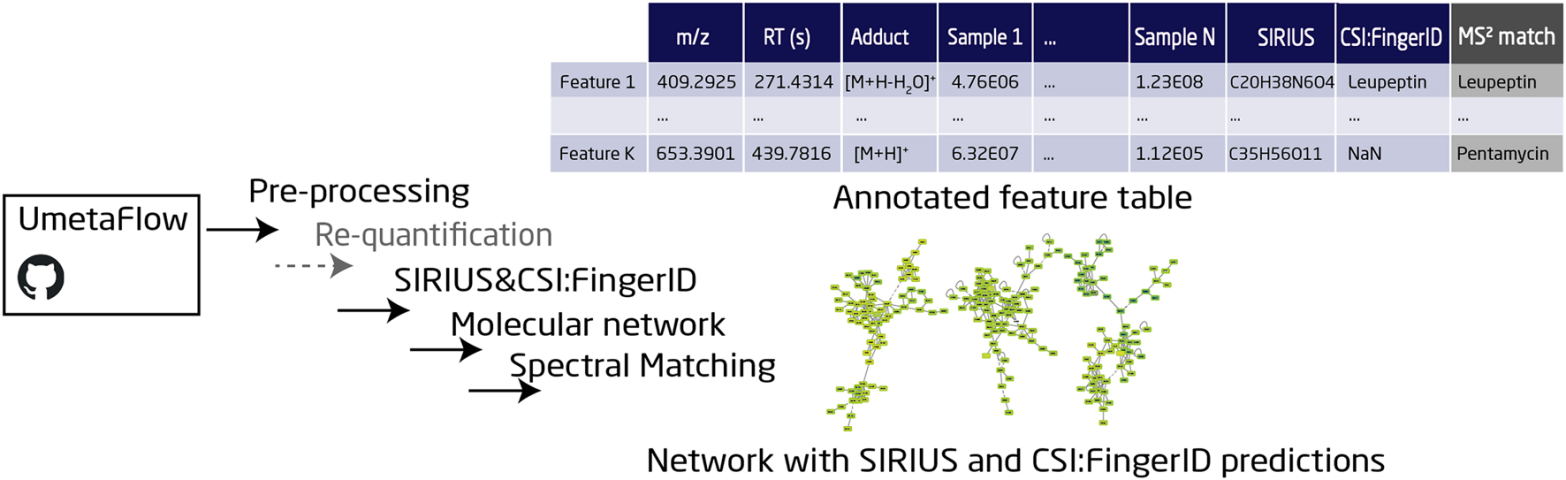

**Supplementary Information:**

The online version contains supplementary material available at 10.1186/s13321-023-00724-w.

## Introduction

Untargeted metabolomics is a rapidly developing field. It is widely used in research on natural products, environmental science, food science, and medicine, such as drug and biomarker discovery [1, 2]. This approach allows for the comprehensive and qualitative or semiquantitative analysis of as many metabolites as possible in a specimen [3, 4].

The sample preparation for metabolomics experiments is inexpensive and easy compared to other *omics* technologies [5] and can be fully automated in many cases [6, 7]. In addition, constant improvements in throughput are taking place, such as UHPLC–MS systems for shorter chromatographic runtimes, as well as chromatography-free direct infusion [8] and flow injection technologies [9, 10]. These techniques allow researchers to perform large-scale studies and achieve acquisition rates of hundreds to thousands of samples per day, with some methods reaching to less than 1 s per sample in acquisition time [5].

These advancements have led to more accessible high-throughput experiments, with numerous institutes moving towards big data. However, even though high-throughput data acquisition is achieved, scaling up data processing and analysis in untargeted metabolomics remains a challenge [11]. Most of the currently available tools are web-based, such as XCMS Online [12] and MetaboAnalyst [13], which can be limiting for sensitive data. Others are restricted to specific operating systems, such as MetAlign 3.0 [14], MS-Dial [15], or have limited scalability when analyzing hundreds or thousands of files, such as MZmine 2 [16].

Here, we report an open-source workflow, UmetaFlow, that applies combinatorial computational algorithms for high-throughput liquid chromatography tandem mass spectrometry (LC–MS/MS) data processing and analysis, using OpenMS [17] 3.0 tools for feature detection, map alignment, adduct annotation, re-quantification and feature linking, spectral matching, and structural and formula predictions via SIRIUS [18] and CSI:FingerID [19]. OpenMS algorithms have been implemented for generating all the files necessary for GNPS Feature-Based Molecular Networking (FBMN) [20] and Ion Identity Molecular Networking (IIMN) [21]. All these steps are complemented with Python scripts for data integration. The workflow is implemented in a workflow manager, Snakemake [22], making it easy to operate in diverse HPC or cloud environments. We evaluated and benchmarked UmetaFlow and demonstrated that it ranks as one of the best tools for feature detection, quantification and marker selection when compared with other untargeted metabolomics software tools, indicating that UmetaFlow can be used as a tool for large-scale metabolomics data processing and analysis.

## Results and discussion

### UmetaFlow overview

UmetaFlow was built for rapid processing of large LC–MS/MS datasets and for that purpose, it is implemented as a Snakemake [23] workflow, allowing high scalability and speed due to parallelization. This version is compatible with macOS and Linux operating systems. In addition, UmetaFlow contains Python bindings to the OpenMS algorithms (pyOpenMS [24]) and other Python modules that are commonly used in data science implemented as Jupyter notebooks. This allows for interactive computing, easy data exploration and visualization, as well as rapid prototyping and implementation of new steps. The python version is compatible with macOS, Linux and Windows operating systems.

UmetaFlow can be divided into four parts: (i) data pre-processing and optional re-quantification that generates a table of metabolic features, (ii) formula and structural predictions, (iii) a GNPS-export step that generates all the files necessary for FBMN [20] and IIMN [21], and (iv) spectral matching. The final output of the workflow is a feature matrix with mass-to-charge (*m/z*), retention time (RT), adduct and peak area (intensity) information of each feature in each input file, as well as fragmented mass spectrum (MS^2^) library matches, and structural and formula prediction annotations. In addition, a GraphML file format originally generated from GNPS is annotated with structural and formula prediction for visual inspection.

Initially, the raw files need to be converted from a vendor-specific format to the open community-driven mzML format. If the data are obtained in profile mode, a peak picking algorithm needs to be applied to convert them to centroided mode for compatibility with the OpenMS algorithms. After centroiding, the ion intensity distribution across *m/z* is reduced to a single point, the peak apex, which leads to significant data reduction. There is an optional initial step in the workflow for file conversion and peak picking of Thermo Fisher raw data through the OpenMS algorithm *FileConverter*. This algorithm uses the ThermoRawFileParser executable (Additional file 1: Figure S1a), which is a straightforward tool compatible with Linux, macOS and Windows operating systems [25]. A popular alternative, which works for other vendor formats as well, is ProteoWizard’s msConvert [26] that can be employed independently (Table 1). ProteoWizard’s msConvert is compatible for Windows and Linux operating systems and thorough documentation is provided at https://proteowizard.sourceforge.io/ [26]. However, vendor software packages should be preferred for centroiding conversion to maintain data integrity.

**Table 1:** Supported vendor-format files for conversion with ProteoWizard and analysis with UmetaFlow

Vendor	Formats
ABI	T2D
Agilent	MassHunter.d
Bruker	Compass.d, YEP, BAF, FID, TDF
Sciex	WIFF/WIFF2
Shimadzu	LCD
Thermo Scientific	RAW
Waters	MassLynx.raw/UNIFI

#### Pre-processing

**Fig. 1:**
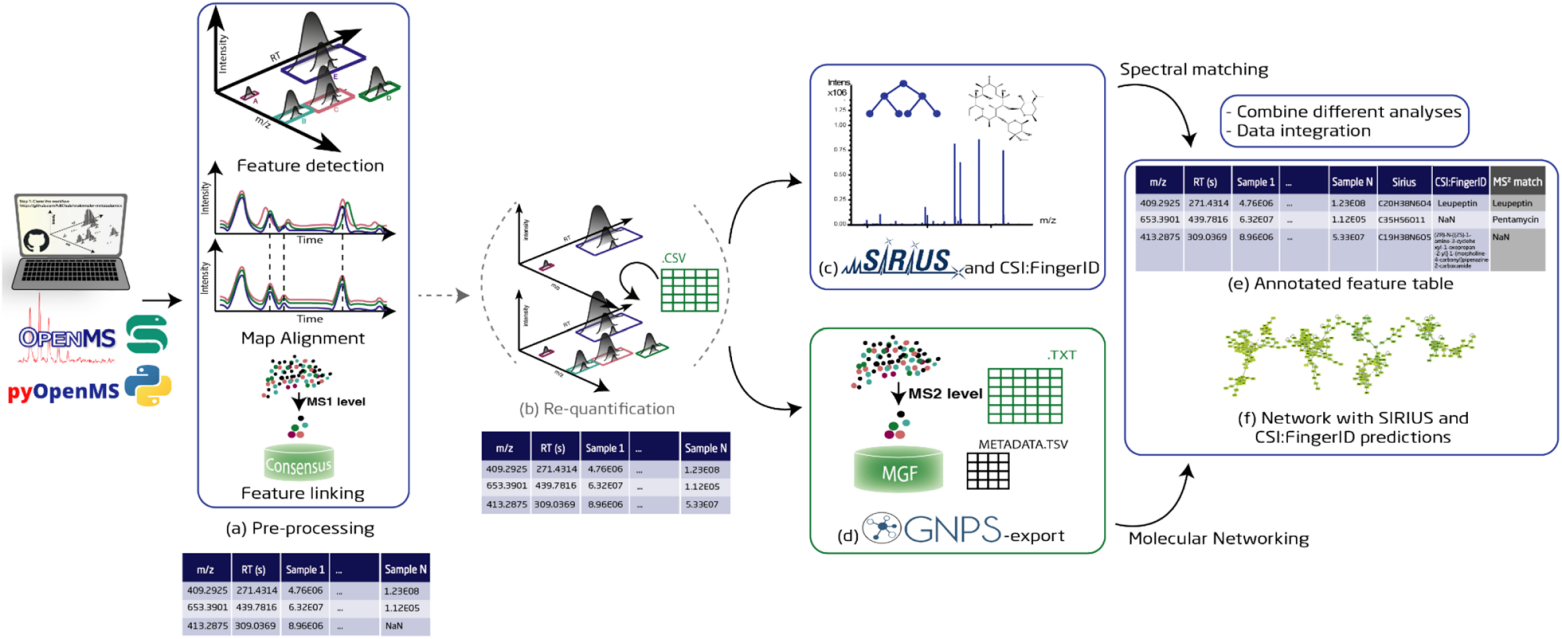
Overview of UmetaFlow. The user can clone UmetaFlow (Snakemake or Jupyter notebook version) from github and follow the step-by-step guide to set it up. **a** The pre-processing step is a set of algorithms that transforms the raw data to a table of metabolic features. One of the most important algorithms of this step is the one for feature detection, that detects mass traces, deconvolutes them and assembles single isotopic mass traces to metabolite features. Map alignment corrects for RT shifts and feature linking connects corresponding features across individual runs. **b** Right after, an optional step for re-quantification of features with missing values can be selected. **c** The generated feature files (re-quantified or not), together with the mzML files, are used as inputs to the SIRIUS executable for formula and structural predictions. **d** The clustered feature files and mzML files are introduced to the *GNPSexport* algorithm to generate all the files necessary for FBMN/IIMN. **e**, **f** The final output of UmetaFlow is a feature matrix and a GraphML network file with MS^2^ library matches, and formula and structural prediction annotations

Pre-processing is a crucial step in metabolomics data mining for transforming the raw data to a table of metabolic features [11]. This part of the workflow uses OpenMS [17] algorithms for feature detection, adduct annotation, feature alignment and clustering (Fig. 1a). Initially, the mzML files are processed with the OpenMS tool *HighResPrecursorMassCorrector*, which corrects for mistakenly assigned precursors of MS^2^ spectra, by selecting the intact mass spectrum level (MS^1^) peak with the highest intensity using RT and mass range information. This algorithm is useful for Data-Dependent Acquisition (DDA) mode, where the most intense ions in a spectrum are selected for fragmentation but can be ignored for other acquisition methods. The feature detection algorithm *FeatureFinderMetabo* detects mass traces of similar *m/z* along the RT dimension, deconvolves (partially) overlapping chromatographic peaks and assembles co-eluting, single mass traces to metabolite features for data reduction [27]. The most important parameters for feature detection are the mass error and noise threshold, defined by the instrument and method that is used to analyze the samples, as well as the peak width, which is directly correlated to the chromatographic system (Additional file 1: Table S1). The feature maps generated by *FeatureFinderMetabo* are containers that include information on each feature, such as *m/z*, RT, charge, and intensity, and are stored as featureXML files, an OpenMS file format for LC–MS data. Here, the user can optionally define blanks, quality controls (QCs) or control samples that will allow for background removal by setting an intensity ratio cutoff. The now filtered featureXML files, together with the corresponding mzML files, are then processed by *HighResPrecursorMassCorrector*, which corrects for mistakenly assigned MS^2^ parent ions to monoisotopic masses. Next, *MapAlignerPoseClustering* [28] performs a linear RT alignment between the featureXML files to correct for any chromatographic RT shifts (Additional file 1: Figure S1c). The file used as a reference for alignment is fetched automatically by the algorithm, if not specified by the user, and it is the file with the highest number of features (e.g., a pooled quality control sample). The mzML files are also introduced to *MapRTTransformer* for RT alignment, using transformation description files (.trafoXML) generated from *MapAlignerPoseClustering* [28]*.* The aligned feature maps are subjected to analysis with *MetaboliteAdductDecharger,* which is used for adduct annotation (Additional file 1: Figure S1e), as well as to convert the charged features to neutral masses, and cluster features that originate from the same metabolite [29]. This algorithm is important for information reduction, formula, and structural predictions, as well as for FBMN. Here, the most important parameter is the list of adducts that are possibly generated by the instrument, in positive or negative ionization, and the probability of their occurrence. *IDMapper* [30] annotates the features that have MS^2^ information to contain necessary metadata for the GNPS-export step. All feature files are finally linked by *FeatureLinkerUnlabeledKD* [31] to match corresponding features over several runs by *m/z* and RT and store all feature information in a single consensus map (Additional file 1: Figure S1g). An optional step allows for filtering features with too many missing values across samples, by a user-defined number that represents the minimum fraction of samples for a feature to be present. Finally, the consensus map is converted to a table of features with information about *m/z*, RT, adduct, as well as presence and intensity of each feature in each input file (Additional file 1: Tables S2, S3 and S4) in a tab-separated format (.tsv).

#### Re-quantification

Untargeted feature detection unavoidably leads to missing values that represent undetected and low-quality features (e.g., missing intensity or mass trace length thresholds). To overcome this problem, a feature intensity value must be imputed, which is implemented in a lot of metabolomics tools by gap filling [11]. Here, we introduce an optional step where if a feature has at least one missing value across all samples, re-quantification is performed to all files (Fig. 1b). In gap filling or any re-quantification step, a secondary feature detection algorithm is used that searches for signals across the mzML files. In UmetaFlow, this step replaces all values across the samples instead of solely the missing one, to maintain comparability of the feature intensities across all samples by using a single quantification algorithm. Using the pre-processed consensus file, a library of features is built from the ones that have at least one missing value along all feature files. This library contains exact mass, charge and RT information and is used as a list of targets for *FeatureFinderMetaboIdent*, a tool that detects and extracts features, commonly used for targeted analysis. The re-quantified feature files are then merged with the previously pre-processed feature files that have no missing values. The merged files are then introduced to *MetaboliteAdductDecharger* (Additional file 1: Figure S1e), *IDMapper*, and finally, to *FeatureLinkerUnlabeledKD* (Additional file 1: Figure S1g) for clustering. An optional step here allows again for filtering features with too many missing values across samples, a number that is user-defined. The resulting file is converted to a tab-separated table (.tsv) of metabolic features. Depending on the dataset to be processed, re-quantification of the feature intensities can be very beneficial for the imputation of missing values, especially when dealing with samples that include identical metabolites in varying concentrations. On the contrary, in a case such as the one of our in-house datasets that were used for validation, where there are very few common metabolites and most true features are present in high concentrations, re-quantification can lead to false positive signals.

#### Formula and structural predictions with SIRIUS and CSI:FingerID

An optional (tentative) identification of the detected features with available fragmentation data is based on *SiriusAdapter,* an OpenMS tool that invokes an externally provided SIRIUS executable (Fig. 1c). SIRIUS [18] generates formula predictions based on scores calculated from MS^2^ fragmentation (ppm error and intensity) and MS^1^ isotopic pattern scores. CSI:FingerID [19] is a web service, which, after the formula predictions are uploaded via the SIRIUS executable, uses those formulas to predict their molecular structure fingerprint using a machine learning approach. The fingerprint is then used to search for matches in structural libraries. Within the *SiriusAdapter* step of UmetaFlow, the user can provide both the mzML and the corresponding pre-processed feature and adduct information (featureXML) as input files to SIRIUS. The algorithm then creates a .ms temporary file (SIRIUS internal format) that is used as an input for the SIRIUS executable, allowing SIRIUS to compute only the MS^2^ spectra that are allocated to a feature, instead of all MS^2^ data. The pre-processed or re-quantified feature matrix is then annotated with the highest ranked predictions from both algorithms using unique feature identifications (Fig. 1e), classified as metabolite annotations level 3, according to the Metabolomics Standard Initiative nomenclature (MSI level 3) [32].

#### Integrating a molecular networking tool: GNPS FBMN/IIMN

One of the most important and widely used tools for molecular networking, annotation and visualization in the metabolomics community is GNPS FBMN [20]. In FBMN, MS^2^ data are searched against publicly available, crowd-sourced spectral libraries and grouped with related molecules, creating networks within a metabolomics experiment. A new workflow, IIMN [21], is also integrated in the GNPS FBMN environment, and allows for connecting and collapsing different adducts of the same feature, improving networks that with sole MS^2^ comparisons often remain unconnected. Our GNPS export sub workflow at the end of the pipeline generates all the files necessary for FBNM and IIMN (Fig. 1d).

FBMN can only analyze features that have associated fragmentation data, so the first step of the GNPS export is to filter the consensus file generated from *FeatureLinkerUnlabeledKD* with the *FileFilter* tool, keeping only features that have MS^2^ information. The consensus file is then introduced to the *GNPSExport* tool together with all the mzML files. The tool is responsible for clustering of the MS^2^ information to a single MGF file, conversion of the consensus file to a Feature Quantification table (TXT) and generation of a comma-separated supplementary table that allows for connecting and collapsing different adducts of the same feature. Additionally, a tab-separated metadata table is created that contains the filename and the map identification number, originally generated from the feature linking algorithm, but the user can manipulate the file to add more information that will provide an advantage to the visual exploration of the network. The OpenMS FBMN workflow in GNPS is still experimental, and the user can submit a job at https://proteomics2.ucsd.edu by choosing the latest release of FBMN.

Once the FBMN/IIMN job is completed, the user can download the data and annotate the GraphML file with SIRIUS and CSI:FingerID predictions to facilitate visual inspection of the network (Fig. 1f).

#### Spectral matching

A common strategy for LC–MS/MS data analysis is to perform spectral matching of the experimental spectra to a library of annotated ones. In untargeted metabolomics, correct spectral annotation helps to avoid rediscovery of already known metabolites. UmetaFlow offers this feature through the OpenMS algorithm *MetaboliteSpectralMatcher*. The user is required to provide a spectral library in an MGF, mzML or MSP file format, which could either be a publicly available spectral library (e.g., GNPS [33] or MassBank of North America [34] that aggregate spectra from various public libraries and user contributions to one location) or an in-house one. The experimental spectral file that is used as an input is the clustered MS^2^ file (MGF) generated from the GNPS export step, and the final output is a feature matrix with MSI level 2 identifications [32] with the highest matching scores (above 60%).

### Workflow implementation

Workflow management tools, such as Snakemake, are ideal for scalability, reproducibility, and easy deployment to different cluster, cloud, or server environments [35]. The workflow engine-enabled version of UmetaFlow is defined by a cascade of integrated rules with specified input and output sets of files. The user has the flexibility to assign a number of threads and achieve parallelization [22] to optimize the runtime. This implementation uses primarily the command line tools of OpenMS 3.0. UmetaFlow is also available in Jupyter notebooks. This version uses Python scripts and, among others, the pyOpenMS 3.0 library. The modular structure of the workflow allows the user to easily add or omit steps, as well as to directly visualize them. Both repositories include a step-by-step guide to set up and run the workflow. Finally, UmetaFlow is also implemented as a web-based Graphical User Interface (GUI) for visualization, parameter optimization and processing of small datasets without the requirement of programming skills. In the GUI, the in silico formula and structural predictions are omitted due to the computational requirements.

### Method evaluation

UmetaFlow was validated and parameter-optimized (Additional file 1: Table S2) with in-house LC–MS–MS/MS data obtained from an UHPLC coupled to a Thermo Orbitrap IDX mass spectrometer from extracts of actinomycete strains that are producing known secondary metabolites, as well as commercial standards. This validation was performed at a pair of Intel(R) Xeon(R) CPU E5-2695 v3 @ 2.30 GHz, with 14 cores per socket and 2 threads per core, with 512 GB of RAM.

The commercial standards that were used for the workflow validation were germicidins A and B, kanamycin, tetracycline hydrochloride, thiostrepton, globomycin, ampicillin and apramycin. The strain extracts that were used for benchmarking were derived from *Streptomyces collinus* Tü 365 (DSMZ 40733) that produces kirromycin and desferrioxamine B [36], *Kutzneria* sp. CA-103260 that produces epemicins A and B [37] and *Streptomyces* sp. NBC 00162 that produces pyracrimycin A [38]. 100% of all expected features were detected in the samples and SIRIUS accurately predicted 76% of all formulas. CSI:FingerID accurately predicted approximately 62% of the structures (Additional file 2: Table S9). SIRIUS supports only singly charged ions with MS^2^ information, so thiostreptone (Additional file 3: Table S10) and epemicin A (Additional file 4: Table S11) could not be computed, since only their doubly charged adduct was fragmented. Finally, the spectral matching step complemented the structural predictions with annotations for the germicidins A and B, kirromycin and siderophores of the desferrioxamine pathway (Additional file 3: Table S10, Additional file 4: Table S11), reaching to a total of 65% accurate structural annotations.

**Fig. 2:**
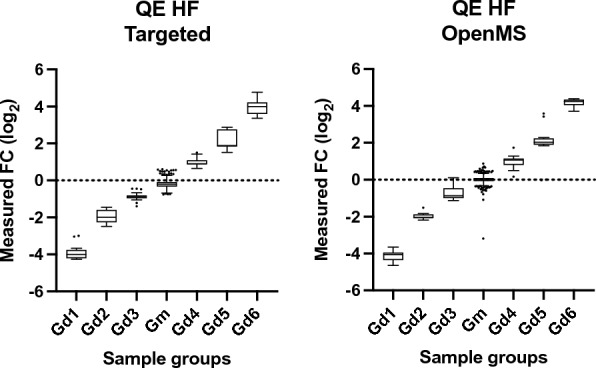
Relative quantification of true features for compounds identified in the standard mixtures. Log-transformed fold changes of features in the benchmark list measured by targeted analysis of the QE HF dataset. Compound concentration ratios of the matrix group (G_m_) and differential groups (G_d1_–G_d6_) are specified in Fig. 1 of the paper by Zhucui Li et al. [39]

**Fig. 3:**
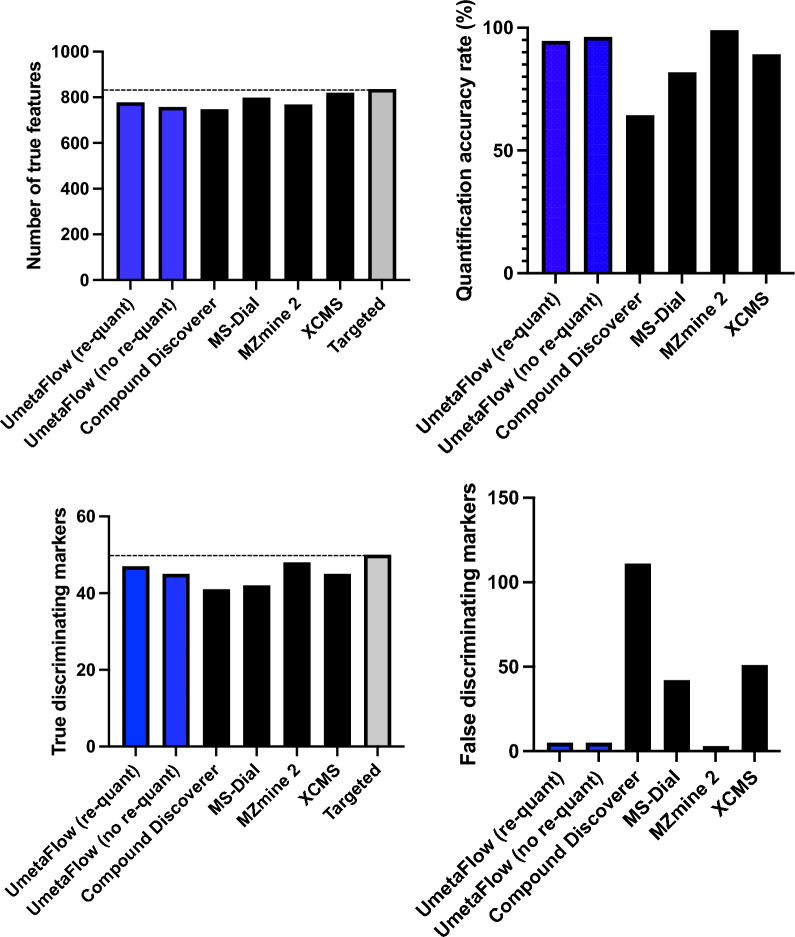
Feature detection, quantification, and marker selection performance between different untargeted metabolomic data processing software using the benchmark dataset MTBLS733 [39]. UmetaFlow is compared with and without the re-quantification step. Refer to Additional file 1: Table S4 for further details

For further validation, the workflow was benchmarked for feature detection, quantification and marker selection using the publicly available Thermo Q Exactive dataset with the MetaboLights accession MTBLS733 that includes two standard mixtures (SA, SB) obtained from *Piper nigrum* extracts with 5 replicates per mixture [39]. Each mixture consists of the same compounds, some of which are in different concentrations. The concentration ratios between the two mixtures define different compound groups (G_m_, G_d1_–G_d6_), as previously described by Zhucui Li et al*.* [39]. In the published research related to the dataset the authors performed targeted analysis using vendor software (refer to the relevant publication for further details) and identified 836 unique features, a number that represents the maximal number of features that can be detected with untargeted software packages and evaluated four untargeted metabolomics processing software (MS-Dial, MZmine 2, XCMS and Compound Discoverer) for feature detection, quantification, and marker selection. To evaluate the software performance quantitatively, all compound-derived true feature fold-changes (SB:SA) were calculated with targeted analysis [39]. Following the author’s directions, and after parameter optimization (Additional file 1: Table S4), UmetaFlow could detect 778 true features, a 93.1% untargeted versus targeted identification rate. Out of all true features detected, 736 were accurately quantified (94.6%). To assess the quantification accuracy of UmetaFlow, the fold-changes of the intensities between the mixtures SA and SB of all true features identified were calculated. Then, those fold-changes (FC) were log-transformed and plotted for comparison of the targeted and untargeted approach and the results indicated high accuracy and low variation between features of the same group (Fig. 2). The dataset included 50 discriminating markers with p values < 0.05 and fold-changes < 0.5 or > 2. UmetaFlow could detect 47 true and only 5 false discriminating markers. The performance of the workflow is significantly enhanced with the re-quantification step, detecting 20 additional true features, as well as detecting 2 more true discriminating markers than if we omit this step (Fig. 3; Additional file 1: Table S5).

**Fig. 4:**
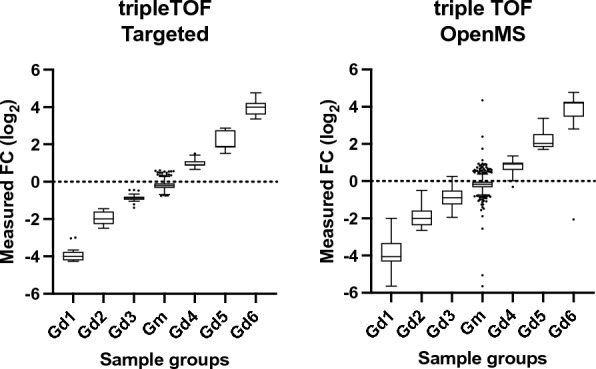
Relative quantification of true features for compounds identified in the standard mixtures. Log-transformed fold changes of features in the benchmark list measured by targeted analysis of the tripleTOF dataset. Compound concentration ratios of the matrix group (Gm) and differential groups (G_d1_–G_d6_) are specified in Fig. 1 of the paper by Zhucui Li et al. [39]

**Fig. 5:**
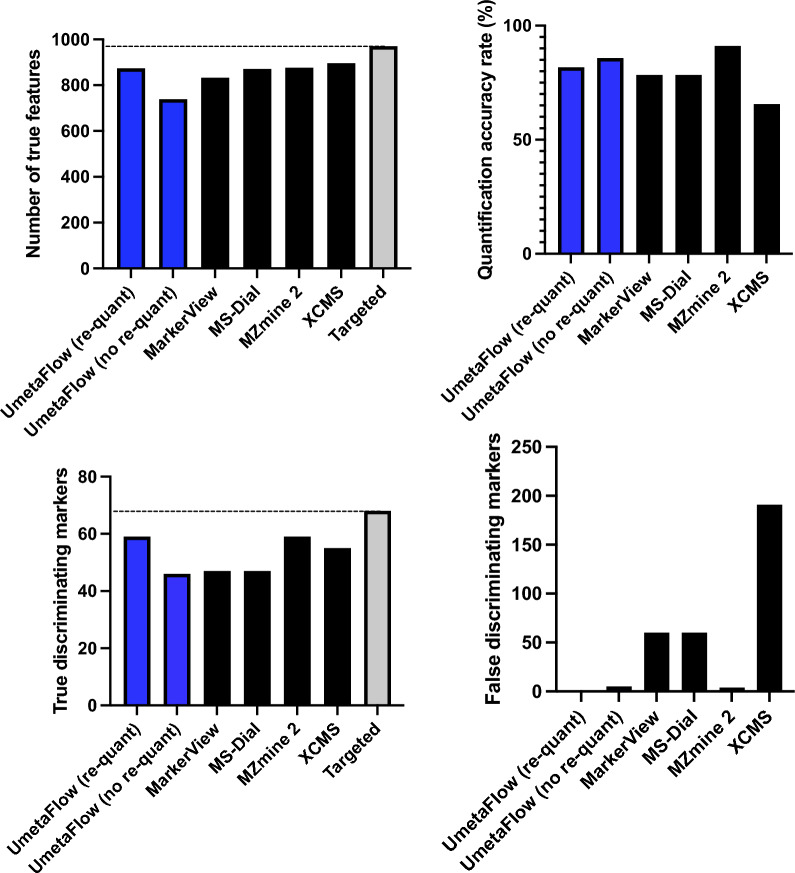
Feature detection, quantification, and marker selection performance between different untargeted metabolomic data processing software using the benchmark dataset MTBLS736 [39]. UmetaFlow is compared with and without the re-quantification step. Refer to Additional file 1: Table S6 for further details

UmetaFlow was further benchmarked using the dataset generated also by Zhucui Li et al. [39], with the MetaboLights accession MTBLS736, analyzed with an AB SCIEX TripleTOF 6600 instrument. After parameter optimization (Additional file 1: Table S6), UmetaFlow could annotate 874 features out of the 970 that were detected using a targeted approach. The workflow could compete with widely used untargeted metabolomics tools (MarkerView, MS-Dial, MZmine2 and XCMS) when compared for feature detection (90.1% true feature ID rate), quantification rate (81.7% accurately quantified features) and discriminating marker selection (59 out of 68 true and 1 false discriminating marker) (Figs. 4, 5, Additional file 1: Table S7). All benchmarking was performed on a MacBook Pro 2020 with 2 GHz Quad-Core Intel Core i5-1038NG7 with 16 GB RAM.

Furthermore, UmetaFlow was validated with the publicly available datasets MTBLS1129 and MTBLS1130 that include patient colon tumors (n = 197) and normal tissues (n = 39) from men and women, to investigate for sex-specific metabolic subphenotypes between cancer tissues on different anatomic locations. The system used for data acquisition in this experiment was a Waters UPLC coupled to a quadrupole time-of flight (QTOF) mass spectrometer and feature detection was performed using XCMS, and specifically the CAMERA package for metabolite annotation. After parameter optimization (Additional file 1: Table S8), UmetaFlow could detect 90% of the features in positive and 100% of the features in negative mode with similar quantification patterns as the published data (Additional file 1: Figure S2a, b) [40].

Finally, UmetaFlow was used to process and annotate a large metabolomic dataset of 1245 raw files acquired from a Thermo Orbitrap IDX instrument and derived from 100 actinomycete strains, grown in three different conditions with three biological replicates per treatment. The parameters used for that dataset were identical to the ones used for the in-house validation data (Additional file 1: Table S2) and they remain as the default parameters of UmetaFlow. Running all 1245 raw files through the pre-processing step was achieved in 1 h 12 m 24 s at a pair of Intel(R) Xeon(R) CPU E5-2695 v3 @ 2.30 GHz, with 14 cores per socket and 2 threads per core, with 512 GB of RAM. Re-quantification took 1 day 17 h 36 min. Acquiring the formula and structural predictions for all files took 9 days 23 h 58 min 16 s and GNPS-export took 29 min and 27 s. The final table consists of 106,578 putative metabolic features. Out of the 56,464 features with MS^2^ information, 1684 (∼ 3%) were annotated with spectral matches (MSI level 2 [32]), 25,976 (∼ 46%) were annotated with formula predictions and 13,722 (∼ 24%) were annotated with both formula and structural predictions (MSI level 3 [32]).

### Opportunities and limitations

UmetaFlow is open-source, fast and scalable, and it allows for the combination of different tools and data integration to facilitate processing and analysis of large untargeted metabolomics datasets. It supports data generated in positive or negative mode and from most instrument types that can be converted to the mzML format (e.g., Thermo Orbitrap, QTOF from various vendors; see Table 1 for a list of supported file formats). By omitting SIRIUS and CSI:FingerID, the user can process low resolution data or data with only MS^1^ information, and by omitting CSI:FingerID and FBMN/IIMN, the user can process sensitive data (e.g., clinical metabolomic datasets). The modular structure of UmetaFlow allows the user to select specific steps to process their dataset with, modify them, but also add supplemental functionality, for example, statistical analysis and visualization steps. The workflow also allows for re-quantification of features that have missing values across all samples, a unique method to impute missing values.

A limitation of UmetaFlow is the requirements for basic programming skills in order to implement and adapt the Snakemake workflow, as well as access to a server, cluster or cloud environment for processing very large datasets, due to storage requirements that exceed specifications of a common PC. However, the user can delete all the interim files at the end of a run. The most computationally demanding process in the workflow is SIRIUS step, which is optional. Nevertheless, for small(er) datasets, the user can run the workflow successfully using a common PC, such like the one used for benchmarking, either through the Snakemake workflow, the Jupyter Notebooks, or the web-based GUI.

## Conclusions

Mass spectrometry data can be numerous and highly complex, creating a need for tools that can analyze large metabolomics datasets. UmetaFlow is a workflow built for automated, high-throughput untargeted mass spectrometry-based metabolomics data processing and analysis using OpenMS algorithms. It allows for fast, scalable, and reproducible analysis through a workflow manager, Snakemake, but also for prototyping or smaller-scale interactive data processing through the Jupyter notebooks. UmetaFlow connects OpenMS, SIRIUS and CSI:FingerID, as well as GNPS FBMN, for processing, annotation, and data interpretation. Here, we show that we have successfully processed 1245 raw files, validated UmetaFlow with in-house data, and benchmarked it using the publicly available datasets MTBLS733 and MTBLS736. UmetaFlow proved to be an efficient tool when compared with widely used untargeted metabolomics software, both in feature detection, quantification, and marker selection. We anticipate that it will become a broadly used tool for research groups that produce large metabolomics datasets or want to analyze large amounts of publicly available data.

## Methods

### Sample preparation

Germicidins A and B were purchased from Cayman Chemical (Ann Arbor, MI, USA) and were dissolved in 1MeOH:1H_2_O:2DMSO to a concentration of 10^–4^ mg/mL. Globomycin from *Streptomyces hagronensis* was purchased from Sigma-Aldrich and was dissolved in 20% v/v DMSO to a concentration of 10^–4^ mg/mL. Anhydrotetracycline hydrochloride was purchased from Cayman Chemical (Ann Arbor, MI, USA) and dissolved in 40% v/v MeOH to a concentration of 12.5·10^–3^ mg/mL. Ampicillin sodium salt, kanamycin sulfate, apramycin sulfate salt and thiostrepton from *Streptomyces azureus* were purchased from Sigma-Aldrich and were dissolved in 40% v/v MeOH to a concentration of 10^–3^, 5.0·10^–3^, 5.0·10^–3^ and 2.5·10^–3^ mg/mL respectively.

*Streptomyces* sp. NBC 00162, *Streptomyces eridani* and *Streptomyces* sp. CA-210063 were grown, extracted, and analyzed as described by Nielsen et al. [38].

*Kutzneria* sp. CA-103260 and *Streptomyces collinus* Tü 365, as well as all strains used for the acquisition of the large-scale study (1245 files) were initially cultivated in 250 mL Erlenmeyer flasks with a stainless-steel spring with 50 mL media ISP2 from in-house frozen stocks. After 48 h of incubation at 30 °C and 200 rpm, the strains were re-inoculated in 24-deep well plates with ISP2 (Yeast extract 4.0 g; Malt extract 10.0 g; Glucose 4.0 g; Distilled water 1000.0 mL; pH 7.2) only for *Streptomyces collinus* Tü 365, DNPM (Dextrin from corn Type I 40.0 g; Bacto soytone 7.5 g; Bacto yeast extract 5.0 g; MOPS 21.0 g; Distilled water 1000.0 mL; pH 7.0) and FPY12 (Fructose 20.0 g; Glucose 10.0 g; Maltose 10.0 g; Bacto peptone 5.0 g; Amicase 5.0 g; Trace elements FPY-12 1 mL; Distilled water 1000.0 mL; pH 7.0; Trace elements FPY-12: FeSO_4_·7 H_2_O 0.5 g; ZnSO_4_·7 H_2_O 0.5 g; MnSO_4_·H_2_O 0.1 g; CuSO_4_·5 H_2_O 0.05 g; CoCl_2_·6 H_2_O 0.05 g; Distilled water 1000.0 mL) media up to a volume of 3.7 mL and optical density (O.D.) of 0.1. After 7 days of incubation at 30 °C and 200 rpm, 200 μL of culture broth per well was transferred in a 96-well Sirocco protein precipitation plate (Waters; 186002448) and positive pressure was applied using a manifold. The supernatant was collected in a 96-well plate. The remaining cells on the filter were disrupted using 3 × 200 μL of methanol that was combined with the supernatant after applying positive pressure. The wells were dried using gentle nitrogen (N2) stream overnight and redissolved in 200 μL of Milli-Q water. Using an Oasis HLB 96-well plate with 60 mg sorbent per well (Waters; 186000679), the samples were purified using 100% v/v MeOH as eluent. The plate was again left overnight under an N_2_ stream, re-dissolved in 150 μL 50% v/v methanol and the samples were transferred to glass sample vials.

### Metabolomic data acquisition with LC–MS–MS/MS

The analysis of the pyracrimycin A-containing samples was performed on a high-resolution mass spectrometer (HRMS) Orbitrap Fusion system, as previously described in Nielsen et al. [38].

The instrumentation that was used for the analysis of the commercial standards, the large-scale study, and the extracts of *Streptomyces collinus* Tü 365 and *Kutzneria* sp. CA-103260 is a Dionex Ultimate 3000 ultra-high-performance liquid chromatography (UHPLC) coupled to a high-resolution mass spectrometer (HRMS) Orbitrap ID-X (ThermoFisher Scientific, Waltham, MA, USA). The UHPLC method used for the analysis was the following: column, Zorbax Eclipse Plus C-18 column (2.1 × 100 mm, 1.8 μm) (Agilent, Santa Clara, CA, USA); column temperature: 40 °C; solvent A (H_2_O buffered with 0.1% HCOOH) and solvent B (CH_3_OH buffered with 0.1% HCOOH); isocratic: 0–0.8 min, 2% B; gradient: 0.8–2.5 min, 2–5% B; gradient: 2.5–10 min, 5–100% B; isocratic: 10–11 min, 100% B; gradient: 11–11.7 min, 100–2% B; isocratic: 11.7–12.7 min, 2% B; flow rate, 0.350 mL/min. The HRMS was performed in positive mode (+ ESI), at 3500 V spray voltage, in the mass range (*m/z*) 100–1500 (70–100 for the DSMZ 40,733 samples) at a resolution of 120 K, RF Lens 60%, and AGC target 400 K. Before analysis, the MS instrument was calibrated using ESI Positive ion Calibration Solution Pierce™ LTQ Velos ESI Positive Ion Calibration Solution. The software Xcalibur 4.2 (Thermo Fisher Scientific Inc.) was used for targeted data analysis.

## Supplementary Information

**Additional file 1. **UmetaFlow: An untargeted metabolomics workflow for high-throughput data processing and analysis. **Figure S1.** A detailed overview of UmetaFlow. **Table S1.** Important instrument, method, and sample-specific parameters for UmetaFlow parameter optimization. **Table S2.** The optimal parameters for OpenMS (UmetaFlow) for feature detection, formula, and structural predictions of the in-house datasets. **Table S3.** Feature detection, structural and formula predictions for pyracrimycin A in *Streptomyces* sp. NBC 00162, *Streptomyces* sp. CA-210063 and *Streptomyces eridani*. **Table S4.** The optimal parameters for OpenMS (UmetaFlow) for feature detection, quantification, and marker selection of the MTBLS733 QE HF dataset. **Table S5**. Feature identification, quantification, and marker selection performance of different untargeted metabolomic data processing software using the benchmark dataset MTBLS733. **Table S6**. The optimal parameters for OpenMS (UmetaFlow) for feature detection, quantification, and marker selection of the MTBLS736 tripleTOF dataset. **Table S7**. Feature identification, quantification, and marker selection performance of different untargeted metabolomic data processing software using the benchmark dataset MTBLS736. **Table S8**. The optimal parameters for OpenMS (UmetaFlow) for feature detection and quantification of the MTBLS1129 and MTBLS1130 dataset. **Figure S1.** Plotted average metabolite intensities in normal and colon cancer tissue samples, detected and quantified with (a) XCMS and (b) UmetaFlow (dataset MTBLS1129).

**Additional file 2: Table S9.** All the raw in-house data were both manually analyzed and through UmetaFlow for method validation.

**Additional file 3: Table S10.** Feature detection, structural and formula predictions for commercial standards germicidins A and B, kanamycin, tetracycline hydrochloride, thiostreptone, globomycin, ampicillin and apramycin.

**Additional file 4: Table S11.** Feature detection, structural and formula predictions for kirromycin and desferrioxamine B from extracts of *Streptomyces collinus* Tü 365 and epemicins A and B from extracts of *Kutzneria sp*. CA-103260.

## Data Availability

MS raw data has been deposited at GNPS (https://gnps.ucsd.edu/) under the following MassIVE ID numbers: - Commercial standards of antibiotics produced by Streptomyces: MassIVE MSV000090047 or at https://zenodo.org/record/6948449. - *Streptomyces collinus* Tü 365 and *Kutzneria* sp. CA-103260 metabolomics files: MassIVE MSV000090048. - *Streptomyces* sp. NBC00162, *Streptomyces eridani* and *Streptomyces* sp. CA-210063 metabolomics files: MassIVE MSV000090049. - Large-scale actinomycete-extract files: MassIVE MSV000090553.

## Code availability

The workflow engine-enabled version of UmetaFlow is freely available under the Apache License 2.0 at https://github.com/biosustain/snakemake_UmetaFlow. The Jupyter notebooks are freely available under the Apache License 2.0 at https://github.com/biosustain/pyOpenMS_UmetaFlow. The web-based GUI is freely available under the Apache License 2.0 at https://github.com/axelwalter/umetaflow-gui.
