# Supplementary material for: UmetaFlow: an untargeted metabolomics workflow for high-throughput data processing and analysis
Source: J Cheminform. 2023 May 12;15:52. doi: 10.1186/s13321-023-00724-w (PMC10176759; doi:10.1186/s13321-023-00724-w)
Supplement: Supplementary file 1 — Additional file 1. UmetaFlow: An untargeted metabolomics workflow for high-throughput data processing and analysis. Figure S1. A detailed overview of UmetaFlow. Table S1. Important instrument, method, and sample-specific parameters for UmetaFlow parameter optimization. Table S2. The optimal parameters for OpenMS (UmetaFlow) for feature detection, formula, and structural predictions of the in-house datasets. Table S3. Feature detection, structural and formula predictions for pyracrimycin A in Streptomyces sp. NBC 00162, Streptomyces sp. CA-210063 and Streptomyces eridani. Table S4. The optimal parameters for OpenMS (UmetaFlow) for feature detection, quantification, and marker selection of the MTBLS733 QE HF dataset. Table S5. Feature identification, quantification, and marker selection performance of different untargeted metabolomic data processing software using the benchmark dataset MTBLS733. Table S6. The optimal parameters for OpenMS (UmetaFlow) for feature detection, quantification, and marker selection of the MTBLS736 tripleTOF dataset. Table S7. Feature identification, quantification, and marker selection performance of different untargeted metabolomic data processing software using the benchmark dataset MTBLS736. Table S8. The optimal parameters for OpenMS (UmetaFlow) for feature detection and quantification of the MTBLS1129 and MTBLS1130 dataset. Figure S1. Plotted average metabolite intensities in normal and colon cancer tissue samples, detected and quantified with (a) XCMS and (b) UmetaFlow (dataset MTBLS1129). [file 13321_2023_724_MOESM1_ESM.docx]

Additional file 1.

# UmetaFlow: An untargeted metabolomics workflow for high-throughput data processing and analysis

Eftychia Eva Kontou^1^, Axel Walter^2,3^, Oliver Alka^2,3^, Julianus Pfeuffer^5,6^, Timo Sachsenberg^2,3^, Omkar S. Mohite^1^, Matin Nuhamunada^1^, Oliver Kohlbacher^2,3,4^ and Tilmann Weber^1^

^1^The Novo Nordisk Foundation for Biosustainability, Technical University of Denmark, Kemitorvet building 220, 2800 Kgs. Lyngby, Denmark

^2^Applied Bioinformatics, Department of Computer Science, University of Tübingen, Sand 14, 72076 Tübingen, Germany

^3^Institute for Bioinformatics and Medical Informatics, University of Tübingen, Sand 14, 72076 Tübingen, Germany

^4^Translational Bioinformatics, University Hospital Tübingen, Schaffhausenstr. 77, 72072 Tübingen, Germany

^5^Visual and Data-Centric Computing, Zuse Institute Berlin, Takustr. 7, 14195 Berlin, Germany

^6^Algorithmic Bioinformatics, Freie Universität Berlin, Takustr. 9, 14195 Berlin, Germany

# Contents:

[Figure S 1. Detailed overview of UmetaFlow. 3](#_Toc130816814)

[Figure S 2. Plotted average metabolite intensities in normal and colon cancer tissue samples, detected and quantified with (a) XCMS and (b) UmetaFlow (dataset MTBLS1129). 13](#_Toc130816815)

[Table S 1. Important instrument, method, and sample-specific parameters for UmetaFlow parameter optimization. 4](#_Toc130816740)

[Table S 2. The optimal parameters for OpenMS (UmetaFlow) for feature detection, formula, and structural predictions of the in-house datasets. 5](#_Toc130816741)

[Table S 3. Feature detection, structural and formula predictions for pyracrimycin A in *Streptomyces* sp. NBC 00162, *Streptomyces* sp. CA-210063 and *Streptomyces eridani*. 6](#_Toc130816742)

[Table S 4. The optimal parameters for OpenMS (UmetaFlow) for feature detection, quantification, and marker selection of the MTBLS733 QE HF dataset. 7](#_Toc130816743)

[Table S 5. Feature identification, quantification, and marker selection performance of different untargeted metabolomic data processing software using the benchmark dataset MTBLS733. 8](#_Toc130816744)

[Table S 6. The optimal parameters for OpenMS (UmetaFlow) for feature detection, quantification, and marker selection of the MTBLS736 tripleTOF dataset. 9](#_Toc130816745)

[Table S 7. Feature identification, quantification, and marker selection performance of different untargeted metabolomic data processing software using the benchmark dataset MTBLS736. 10](#_Toc130816746)

[Table S 8. The optimal parameters for OpenMS (UmetaFlow) for feature detection and quantification of the MTBLS1129 and MTBLS1130 dataset. 11](#_Toc130816747)


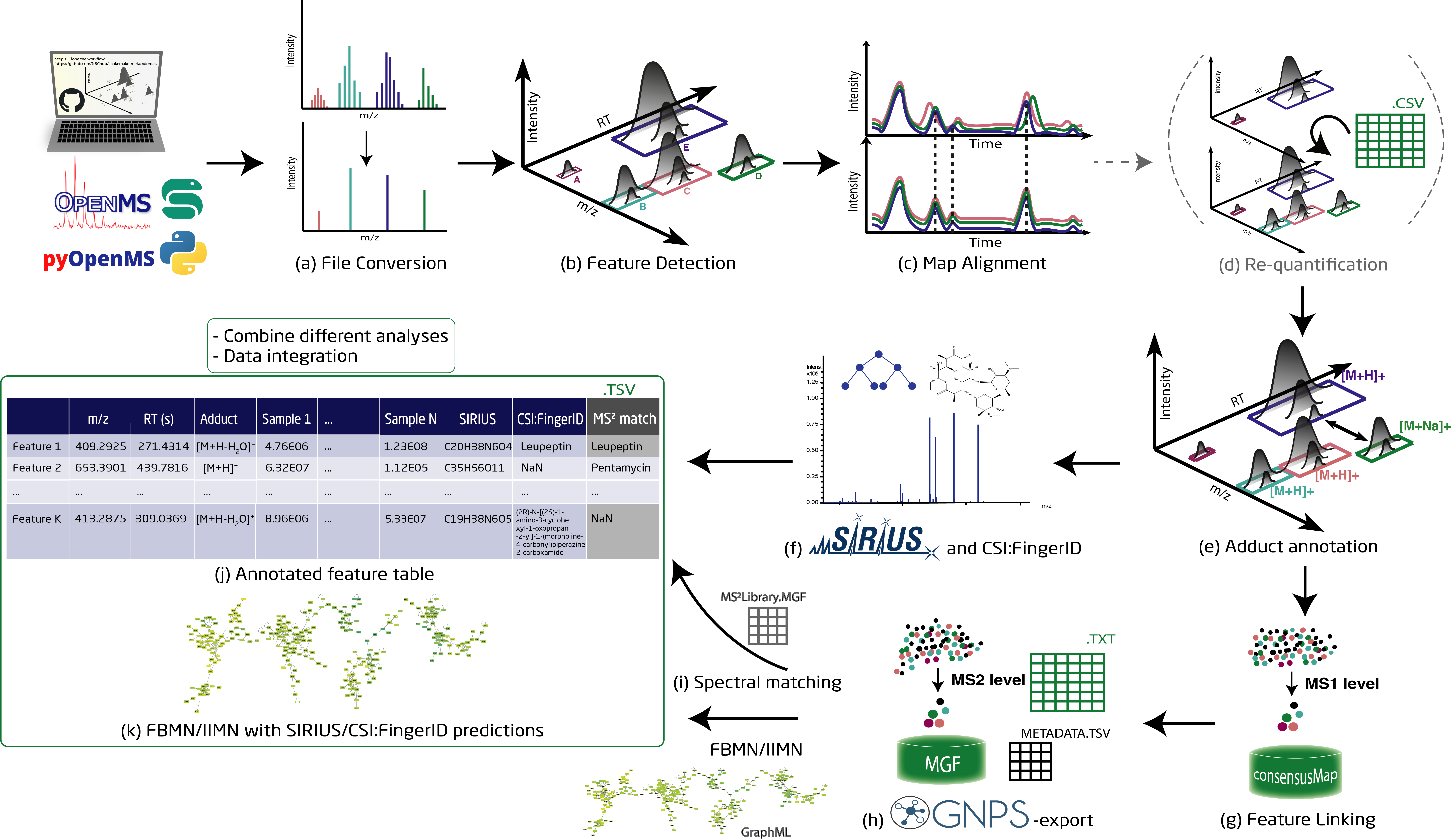


Figure S 1. Detailed overview of UmetaFlow. The user can clone UmetaFlow (Snakemake or Jupyter notebook version) from GitHub and follow the step-by-step guide to set it up. (a) The first step is the file conversion from Thermo RAW files to centroid mzML using the ThermoRawFileParser executable (optional). (b) Next, the feature detection set of algorithms detects mass traces, deconvolves them and assembles single isotopic mass traces to metabolite features. (c) The resulting feature files, as well as the mzML files, are aligned to correct for any linear retention time shifts. (d) The optional re-quantification step of features with missing values can be selected. (e) The features are annotated with adducts, linking features that belong to the same metabolite. (f) The generated feature files (re-quantified or not), together with the mzML files, are introduced to SIRIUS and CSI:FingerID for formula and structural predictions. (g) The feature files are stored to a single consensus map and together with the mzML files they are introduced to the (h) GNPSexport algorithm to generate all the files necessary for FBMN/IIMN. (i) The clustered MS^2^ data (MGF format) are matched with a local library with annotated MS^2^ spectra. (j) The final output of UmetaFlow is a feature matrix and (k) a GraphML network file with MS^2^, and formula and structural prediction annotations.

Table S 1. Important instrument, method, and sample-specific parameters for UmetaFlow parameter optimization.

| Algorithm | Parameters | Description |
| --- | --- | --- |
| FeatureFinderMetabo (FFM)  (Feature finding algorithm) | noise_threshold_int | Intensity threshold below which peaks are regarded as noise. |
|  | remove_single_traces | Remove unassembled traces (traces without satellite isotopic patterns). |
|  | chrom_fwhm | Expected chromatographic peak width. |
|  | mass_error_ppm | Allowed mass deviation, depending on the instrument mass accuracy. |
|  | min_trace_length | Minimum expected length of a mass trace (in seconds). |
| MetaboliteAdductDecharger  (Adduct annotation algorithm) | potential_adducts | Change according to common adducts generated by the instrument. |
| FeatureFinderMetaboIdent  (re-quantification algorithm) | mz_window | *m/z* window size for chromatogram extraction. Instrument-specific on *m/z* accuracy. |
|  | rt_window | RT window size (in sec.) for chromatogram extraction. Chromatographic shift related. |
| FeatureLinkerUnlabeledKD  (Feature linking algorithm) | link:mz_tol | *m/z* tolerance for linking features from different files. Instrument specific on *m/z* accuracy. |
| MapAlignerPoseClustering  (RT alignment algorithm) | distance_mz:max_difference | Never pair features with larger *m/z* distance. Instrument specific on *m/z* accuracy. |

Table S 2. The optimal parameters for OpenMS (UmetaFlow) for feature detection, formula, and structural predictions of the in-house datasets.

| Parameters | Orbitrap IDX |
| --- | --- |
| FFM/Noise threshold intensity | 10,000 |
| FFM/Remove single mass traces | True |
| FFM/Chromatogram FWHM | 0.5 |
| FFM/Mass error | 10 (ppm) |
| SIRIUS/filter_by_num_masstraces | 2 |
| SIRIUS/elements_enforced | CHN[15]OS[4]Cl[2]P[2] |
| SIRIUS/db | none |

Table S 3. Feature detection, structural and formula predictions for pyracrimycin A in Streptomyces sp. NBC 00162, Streptomyces sp. CA-210063 and Streptomyces eridani. Here, there are quite a lot of identical features and MS^2^ spectra matching to pyracrimycin A along the retention time dimension in the Streptomyces sp. CA-210063 sample. The red color indicates wrong adduct annotation, formula, or structural prediction. The spectral matching column is not presented because it did not generate results for the specific features.

| SIRIUS | CSI_name | CSI_smiles | m/z | RT (s) | Adduct | *Streptomyces* sp. CA-210063 | *Streptomyces eridani* | *Streptomyces* sp. NBC00162 |
| --- | --- | --- | --- | --- | --- | --- | --- | --- |
| C7H10N2O | 3-Methoxy-2,5-dimethylpyrazine | CC1=CN=C(C(=N1)OC)C | 139.0864555 | 154.666 | [M+H]^+^ |  | 3.58E+06 |  |
| C7H10N2O | 5-Amino-2-methoxy-4-picoline | CC1=CC(=NC=C1N)OC | 139.0865397 | 76.179 | [M+H^]+^ | 2.73E+07 |  |  |
| C7H10N2O | p-Diaminoanisole | COC1=C(C=CC(=C1)N)N | 139.0864509 | 108.441 | [M+H]^+^ | 7.33E+08 |  | 3.63E+07 |
|  |  |  | 139.0865178 | 262.877 | [M+H]^+^ | 2.82E+07 |  |  |
| C7H7NO | zlchem 463 | C1=CC(=CC(=C1)N)C=O | 139.0865166 | 253.029 | [M+4H+N]^+^ | 3.71E+07 |  |  |
| C7H7NO | Benzamide | C1=CC=C(C=C1)C(=O)N | 139.0865163 | 280.211 | [M+4H+N]^+^ | 7.84E+07 |  |  |

Table S 4. The optimal parameters for OpenMS (UmetaFlow) for feature detection, quantification, and marker selection of the MTBLS733 QE HF dataset.

| Parameters | QE HF (Thermo) |
| --- | --- |
| FFM/Noise threshold intensity | 50,000 |
| FFM/Minimum trace length | 0.2 |
| FFM/Remove single mass traces | False |
| FFM/Chromatogram FWHM | 0.2 |
| FFM/Mass error | 10 (ppm) |
| Requantification/Extract mz window | 10 (ppm) |
| Requantification/Extract RT window | 30.0 s |

Table S 5. Feature identification, quantification, and marker selection performance of different untargeted metabolomic data processing software using the benchmark dataset MTBLS733. UmetaFlow is compared with and without the re-quantification step.

|  |  | Total features | Consensus features | True features | True feature ID rate (%) | Accurately quantified true features | Quantification accuracy rate (%) | True discriminating markers | False discriminating markers |
| --- | --- | --- | --- | --- | --- | --- | --- | --- | --- |
| Targeted |  | - | - | 836 | - | 836 | 100 | 50 | 0 |
| Untargeted | UmetaFlow (re-quantification) | 25,385 | 16,874 | 778 | 93.1 | 736 | 94.6 | 47 | 5 |
|  | UmetaFlow  (w/o re-quantification) | 35,795 | 8,294 | 758 | 90.7 | 730 | 96.3 | 45 | 5 |
|  | Compound Discoverer | 10,525 | 10,525 | 748 | 89.5 | 482 | 64.4 | 41 | 111 |
|  | MS-Dial | 21,545 | 17,726 | 799 | 95.6 | 654 | 81.9 | 42 | 42 |
|  | MZmine 2 | 20,021 | 18,871 | 769 | 92.0 | 761 | 99.0 | 48 | 3 |
|  | XCMS | 35,215 | 30,680 | 820 | 98.1 | 731 | 89.2 | 45 | 51 |

Table S 6. The optimal parameters for OpenMS (UmetaFlow) for feature detection, quantification, and marker selection of the MTBLS736 tripleTOF dataset.

| Parameters | tripleTOF (SCIEX) |
| --- | --- |
| FFM/Noise threshold intensity | 1.0 |
| FFM/Minimum trace length | 0.2 |
| FFM/Remove single mass traces | False |
| FFM/Chromatogram FWHM | 0.2 |
| FFM/Mass error | 10 (ppm) |
| Requantification/Extract mz window | 10 (ppm) |

Table S 7. Feature identification, quantification, and marker selection performance of different untargeted metabolomic data processing software using the benchmark dataset MTBLS736. UmetaFlow is compared with and without the re-quantification step.

|  |  | Total features | Consensus features | True features | True feature ID rate (%) | Accurately quantified true features | Quantification accuracy rate (%) | True discriminating markers | False discriminating markers |
| --- | --- | --- | --- | --- | --- | --- | --- | --- | --- |
| Targeted |  | - | - | 970 | - | 970 | 100 | 68 | 0 |
| Untargeted | UmetaFlow (re-quantification) | 69,242 | 27,688 | 874 | 90.1 | 714 | 81.7 | 59 | 1 |
|  | UmetaFlow  (w/o re-quantification) | 155,331 | 24,451 | 739 | 76.2 | 634 | 85.8 | 46 | 5 |
|  | MarkerView | 20,000 | 9718 | 833 | 85.9 | 683 | 78.4 | 47 | 60 |
|  | MS-Dial | 26,185 | 15,582 | 871 | 89.8 | 683 | 78.4 | 47 | 60 |
|  | MZmine 2 | 24,472 | 23,677 | 876 | 90.3 | 798 | 91.1 | 59 | 4 |
|  | XCMS | 28,168 | 25,386 | 896 | 92.4 | 588 | 65.6 | 55 | 191 |

Table S 8. The optimal parameters for OpenMS (UmetaFlow) for feature detection and quantification of the MTBLS1129 and MTBLS1130 dataset.

| Parameters | QTOF (Waters) |
| --- | --- |
| FFM/Noise threshold intensity | 1.0e03 |
| FFM/Remove single mass traces | False |
| FFM/Mass error | 10 (ppm) |

Figure S 2. Plotted average metabolite intensities in normal and colon cancer tissue samples, detected and quantified with (a) XCMS and (b) UmetaFlow (dataset MTBLS1129).

a

b
